# Fusion of visible and thermal images improves automated detection and classification of animals for drone surveys

**DOI:** 10.1038/s41598-023-37295-7

**Published:** 2023-06-27

**Authors:** B. Santhana Krishnan, Landon R. Jones, Jared A. Elmore, Sathishkumar Samiappan, Kristine O. Evans, Morgan B. Pfeiffer, Bradley F. Blackwell, Raymond B. Iglay

**Affiliations:** 1 Geosystems Research Institute, Mississippi State University, Mississippi State, Mississippi State, MS 39762 USA; 2 Department of Wildlife, Fisheries, and Aquaculture, Mississippi State University, Box 9690, Mississippi State, MS 39762 USA; 3 Department of Forestry and Environmental Conservation, Clemson University, Clemson, SC 29634 USA; 4 U.S. Department of Agriculture, Animal and Plant Health Inspection Service, Wildlife Services, National Wildlife Research Center, Ohio Field Station, Sandusky, OH 44870 USA

**Keywords:** Ecology, Image processing, Machine learning

## Abstract

Visible and thermal images acquired from drones (unoccupied aircraft systems) have substantially improved animal monitoring. Combining complementary information from both image types provides a powerful approach for automating detection and classification of multiple animal species to augment drone surveys. We compared eight image fusion methods using thermal and visible drone images combined with two supervised deep learning models, to evaluate the detection and classification of white-tailed deer (*Odocoileus virginianus*), domestic cow (*Bos taurus*), and domestic horse (*Equus caballus*). We classified visible and thermal images separately and compared them with the results of image fusion. Fused images provided minimal improvement for cows and horses compared to visible images alone, likely because the size, shape, and color of these species made them conspicuous against the background. For white-tailed deer, which were typically cryptic against their backgrounds and often in shadows in visible images, the added information from thermal images improved detection and classification in fusion methods from 15 to 85%. Our results suggest that image fusion is ideal for surveying animals inconspicuous from their backgrounds, and our approach uses few image pairs to train compared to typical machine-learning methods. We discuss computational and field considerations to improve drone surveys using our fusion approach.

## Introduction

Drones (small unoccupied aircraft systems or UAS) are increasingly used for monitoring animals, offering multiple advantages, including time or cost savings, increased safety over occupied aircraft, and more accurate counts than traditional ground-based methods^1–4^. Drones can also quickly collect large amounts of data at fine spatial, spectral, and temporal resolutions. Visible (e.g., red, 650 nm; green, 550 nm; blue, 450 nm) or thermal (7.5–14 µm) cameras, yield image or video data that can be used to detect and classify animals either manually or autonomously by computers^5–8^. Human detection (i.e., finding an animal) or classification (i.e., identifying an animal) can be tedious, costly, and error-prone leading to lower detection rates and misclassification errors^5,9,10^. Some biologists have used crowd sourcing or citizen science efforts to manually detect and classify animals in images^11,12^, while others are turning to automated detection and classification through machine learning, specifically deep learning methods like convolutional neural networks (CNN) and computer vision^6,13–15^.

Automated detection and classification have been found to be more accurate and time efficient than human detection and classification in aerial images^5,7,9^, including citizen science approaches^10,14,16^. Recent work has focused on deep learning methods such as CNN to detect and classify animals in images^13–16^. However, detection and classification can often be difficult, not only because of the absence of prominent distinguishing features^13^, but also uncontrollable factors such as obstruction from overhead vegetation or neighboring animals^6,17^, confusion between animals and associated ghost images created from the mosaicking process^14^, or a lack of contrast between animals of interest and their background (e.g., cryptic in visible imagery or homogenous temperature in thermal imagery; reviewed in^6^).

High success or accuracy of machine learning in computer vision stems from the availability of substantial of labelledimages^18^. Image labelling or annotation is the process of marking areas in an image (usually with a rectangular box, referred to as a ‘bounding box’) with class labels such as animal species. However, large, open access databases of annotated animal images from aerial perspectives are lacking to train computer vision algorithms to detect and classify animals in drone images. To our knowledge, primary available databases are those associated with single studies, which often limit the diversity of species, environments, animal poses, and background and color variability surrounding animals captured^14^. In this low-sample learning scenario, typical image augmentation techniques (e.g., rotation, scaling, etc.) often do not account for texture variability in the object and background^19^. Meanwhile, computer vision algorithms are tasked with evaluating entire drone images, not only the cropped regions, which only contain one animal each. Further, unlike camera trap images^20^, the background is constantly changing among drone images, which makes learning the animal features among various backgrounds critical for efficient performance of animal detection and classification in drone images, whether manually or with computer vision.

Combining information from multiple sensors (e.g., visible and thermal images) offers another approach to improve the distinguishability of an animal from the background^21^. Image fusion is the process of combining corresponding image information on the scale of each pixel or group or pixels from multiple image modalities (e.g., visible and thermal images) to generate a single image containing more information than either source image alone^22^. Processing the ‘fused’ image instead of the individual visible or thermal images has shown improved performance among multiple computer vision problems including automated detection and classification in terrestrial imagery^21,23^. Unlike deep learning engines, which use only visible imagery to achieve similar results^24^, large quantities of correctly annotated data and ample training resources are often not required for fusion methods.

Fusion of thermal and visible images has been used for a variety of applications, including autonomous driving (especially in low light situations), surveillance, defect identification, electronic testing, medical imaging, and remote sensing^25^. Fortuitously, many newer drone models and associated imaging sensors are equipped with dual thermal/visible cameras capable of collecting both image types simultaneously (e.g., DJI Zenmuse XT2). To date, however, image fusion has only been tested in four studies involving animals, including one study identifying animals posing hazards to autonomously driven vehicles^26^, and another to identify livestock from unoccupied ground vehicle^27^. Two additional studies pioneered fusion approaches to identify animal species from drone images based on combining visible and thermal data to detect captive white-tailed deer (*Odocoileus virginianus*^17^) and a few individuals of four species in zoo enclosures^28^. However, large advances in both commercially available drone sensors and computer vision approaches since these studies provide opportunities to improve on their methodology and results.

Fusion of visible and thermal information in drone imagery to automatically detect and classify animals is a promising yet relatively untested avenue for improving the efficiency of drone surveys, particularly when few images are available for training machine learning algorithms^6^. We evaluated the performance of image fusion of thermal and visible information in drone imagery for three animal species: white-tailed deer, domestic cow (*Bos taurus*), and domestic horse (*Equus caballus*). We compared performance metrics of eight image fusion methods in two deep learning classification networks to automatic classification of test species using visible and thermal images alone. Finally, we discuss computational and field considerations in using our fusion approach to maximize the information gained from drone surveys that could be scaled up across a range of animal species and conditions.

## Methods

### Study area

We collected study images among research facilities located at Mississippi State University, USA in 2021 and 2022 (Supplementary Fig. S1 online). We used deer enclosures on the Forest and Wildlife Research Center (33.439 N, −88.791 W) and paddocks on the H. H. Leveck Animal Research Center (33.436 N, −88.797 W), which is part of the Mississippi Agricultural and Forestry Experiment Station.

### Drone data collection

We captured images of white-tailed deer, domestic cattle, and domestic horse (hereafter deer, cow, and horse, respectively), during diurnal hours using a DJI Zenmuse XT2 (8-mm visible and 25-mm thermal lenses) mounted on a DJI Matrice 200 V2 quadcopter (SZ DJI Technology Co., Ltd., Shenzen, China). Flights were conducted by a Part 107 certified remote pilot (FAA 2016) through the DJI Pilot app on a Samsung T500 tablet (Samsung Electronics America, Inc., Ridgefield Park, New Jersey, USA) with the sensor in nadir position (i.e., 90° or straight down). We used either autonomous flights with a lawnmower pattern with > 50% overlap, or conducted manual flights, at 30–121 m altitude above ground level (6.9–28.4 mm Ground Sampling Distance) to simultaneously collect visible and thermal images during missions associated with other UAS efforts. Collected images were stored in the open-source Aerial Wildlife Image Repository-AWIR (https://projectportal.gri.msstate.edu/awir/). Methods were approved by NWRC IACUC Number QA-3267 and MSU IACUC (i.e., methods reviewed but no protocol necessary), and we followed all relevant guidelines and regulations for data collection.

### Image processing

Input data totaled 164 images, including 68 images with 265 cows, 53 images with 77 deer, and 43 images with 136 horses (Table 1). From collected images, we first identified image pairs in which one or more animals were present. To maximize variation of animals in our dataset for training fusion methods, we omitted sequential images of the same animal without pose variation in the same series of images on the same day. Second, we annotated images by manually drawing bounding boxes around each animal object and labelling them to species. Finally, because thermal (640 × 512 pixels) and visible (4000 × 3000 pixels) images were of different sizes, we aligned the smaller thermal images by upscaling and translating them with the corresponding region of the larger visible image using image registration procedures^29–31^ (see Supplementary Information Sect.  1.1 online) to obtain a final pixel size of 1792 × 1434 pixels for all images.

**Table 1:** Numbers of images (Images) and individual animal objects (Objects) within images used for training, validation, and testing fusion methods for automated classification of domestic cattle (*Bos taurus*), white-tailed deer (*Odocoileus virginianus*), and domestic horses (*Equus caballus*) from images taken by a drone (unoccupied aircraft system or UAS).

Category	Cow	Deer	Horse
Training images	51	38	28
Training objects	218	61	88
Validation images	4	5	5
Validation objects	16	5	18
Test images	13	10	10
Test objects	31	11	30

### Image fusion

**Figure 1:**
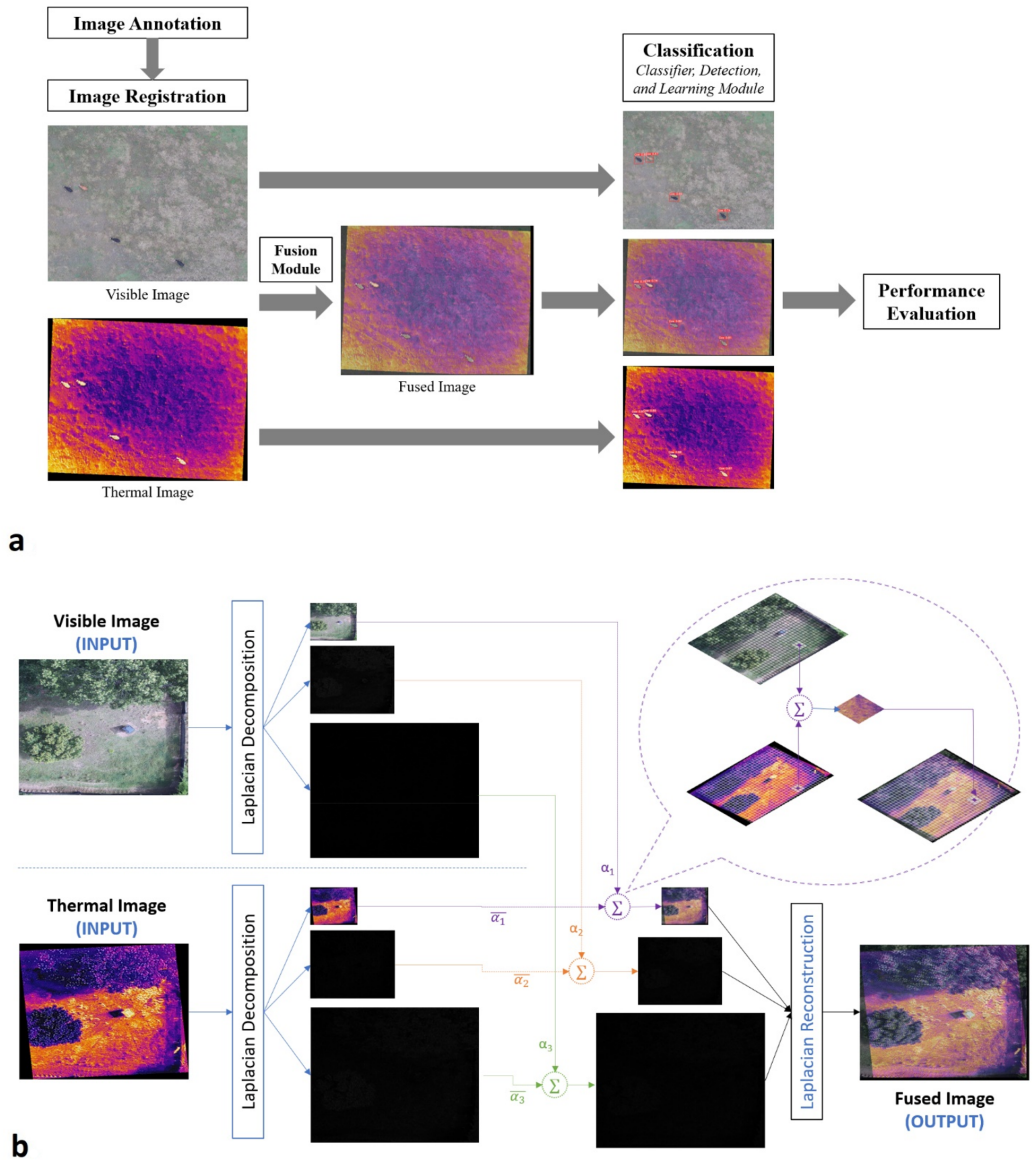
Workflow for fusion of thermal and visible images for learning-based animal object detection and classification from drone (unoccupied aircraft system or UAS) imagery (**a**) and a depiction of the Lalacian fusion algorithm used showing the layer coefficients and block wise coefficients combining in the approximation layer as well as the reconstructed image (**b**).

After respective pairs of visible and thermal images were acquired and registered, their information was combined through fusion before splitting the dataset for training and classification (Fig. 1a, see Supplementary Information Sect. 1.2 online). The image fusion portion of our process followed three general steps for visible and thermal images in each pair: (1) transform both images to a different feature space, (2) merge the information from both images to create the fused image in the transformed feature space, and (3) reconstruct the fused image by an inverse transform of merged information. For some fusion approaches (optimization-based), transforms during the first step were not applied and only the second step occurred.

**Figure 2:**
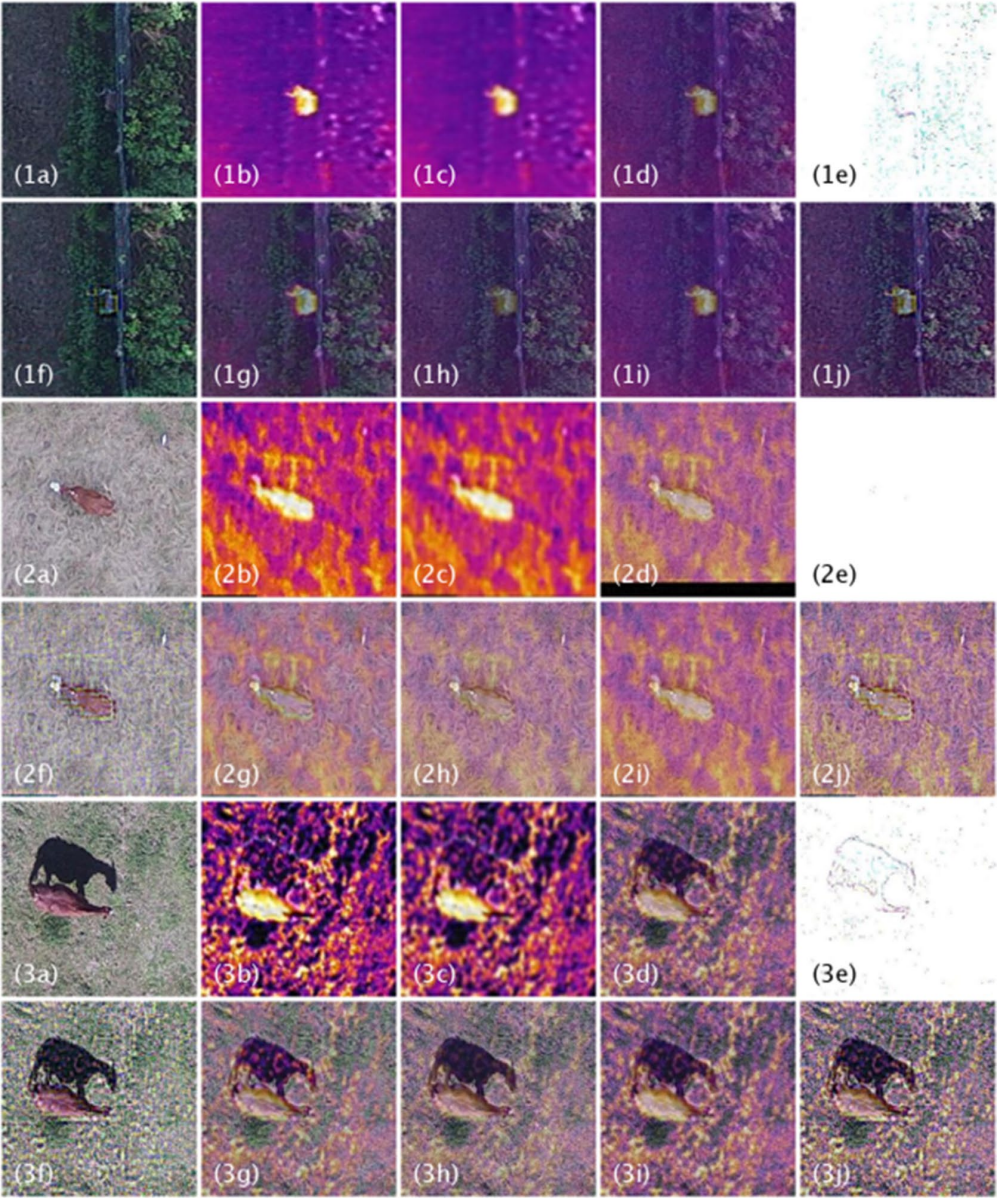
Comparison of aerial imagery captured by drone (unoccupied aircraft system or UAS) containing white-tailed deer (*Odocoileus virginianus*; series 1), cow (*Bos taurus*; series 2), and horse (*Equus caballus*; series 3) among visible (**a**) and thermal (b) images and eight fusion methods: guided filter (**c**), Laplacian (**d**), SVD (**e**), sparse (**f**), gradient (**g**), TVM (**h**), WL + Swarm (**i**), and WL + TVM (**j**).

To compare the performance of fusion methods to visible or thermal images to detect and classify animals, we tested eight different image fusion methods: four multi-resolution-based approaches, two optimization-based approaches, and two hybrid approaches. We evaluated the following multi-resolution-based approaches: (1) guided filter, (2) Laplacian/Gaussian pyramid (LP), (3) singular-value decomposition (SVD), and (4) sparse representation (sparse; Fig. 2). Multi-resolution approaches transform the original image in multiple scales, where the amount (resolution or number of pixels) and type (approximation, detail) of information differs in each scale (Fig. 1b). Image fusion was then performed in each corresponding scale in the transform space. We also used two optimization-based fusion methods, Gradient and total variation distance (TVM). Optimization-based approaches conduct fusion of visible and thermal images at the pixel level to optimize a chosen criterion without image transformation. Finally, we used two hybrid fusion methods, a wavelet (WL) plus TVM hybrid approach (WL + TVM) and a WL plus swarm hybrid approach (WL + Swarm). Hybrid approaches first transform the image to a multi-resolution representation and then fuse in the transform space based on an optimization criterion, combining some aspects of both multi-resolution and optimization-based approaches.

### Object detection and classification

You Only Look Once (YOLO) is a popular deep learning-based object detection architecture. YOLO’s key idea is to frame object detection as a regression problem, thus predicting bounding boxes and confidence probabilities in a single pass of the image through the neural network. This one-shot algorithm excels both in accuracy and speed. By considering multiple scales and aspect ratios, YOLO can handle objects of various sizes and shapes efficiently. YOLO has undergone several iterations with latest being YOLOv8. The newer YOLOv7 also provides focal loss, ideal for identifying small objects but also computationally intense compared to YOLOv5. After image fusion, we used YOLOv5^32,33^ and YOLOv7^34^ to automatically detect and classify objects. Objects evaluated in this study were annotated areas in images. Objects included animals (i.e., animal objects) or non-animals such as annotations by us or incorrect annotations by YOLO architectures (i.e., false positives; see Evaluation criteria for more information). We annotated all animal objects in our image dataset with ground-truth bounding boxes.

We used an approximate 70–10–20% split of images for training–validating-testing classification architectures among species. The same training–validating-testing data were used between classification networks to allow for cross-comparison with our annotated animal objects. However, in the testing procedure, we provided full images without annotations, which often contained multiple animal objects. The trained architectures then created bounding boxes around objects detected as animals and provided the classification of each object in the output. Both architectures were trained and tested on Google Colab Pro using GPU acceleration, using at least 100 Intel Xeon CPUs with a frequency of 2.30 GHz, allocating an average 38 GB of GPU RAM. Because most drone images are larger (our final images were 1792 × 1434 pixels) to cover a large field of view for survey and other applications than typical segmented images processed by these architectures (256 × 256 pixels), our larger image sizes and different network architectures on the same computing resources permitted a maximum batch size of 12 for YOLOv7 and 16 for YOLOv5, using 135 and 100 epochs, respectively. Additional details are available in Supplementary Information Sect. 1.3 online.

### Evaluation criteria

We evaluated the performance of fusion methods based on metrics of (1) animal object quality and (2) classification accuracy (additional details available in Supplementary Information Sect.  1.4 online). We used our annotated animal objects in our test image dataset to evaluate animal object quality metrics of entropy, mutual information, and a gradient-based Petrovic metric. Entropy is the average number of bits per pixel needed to represent an image region^35^, or the animal object within a bounding box for our purposes. A higher value of entropy implies a larger amount of information in the image region, which typically improves differentiation of animal objects from their respective backgrounds. Mutual information (bits per pixel) represents the amount of information transferred from an individual image (visual or thermal) to the fused image. The two values of mutual information from the visual and thermal images were summed; higher values were preferred and indicated that a larger amount of useful information was transferred to the fused image compared to lower values^35^. The gradient-based Petrovic metric is a unitless measure of edge preservation ranging from 0 to 1^36,37^. Values closer to 1 indicated higher preservation of edge information compared to values closer to 0 because the visual perception of an object is first based on identifying its edges. Thus, details in the pixels at the edges of an object contain most of the information comprising its shape compared to middle regions^22^, as is the case for animals in our drone images. To visualize patterns and compare the performance of fusion metrics, we created plots of all three metrics of image quality for each animal object (entropy) or object pair (mutual information, Petrovic metric) in our dataset. Because these metrics are specific to their respective backgrounds within bounding boxes, we represented them as values for individual animal objects and did not average them across fusion methods for comparison.

**Figure 3:**
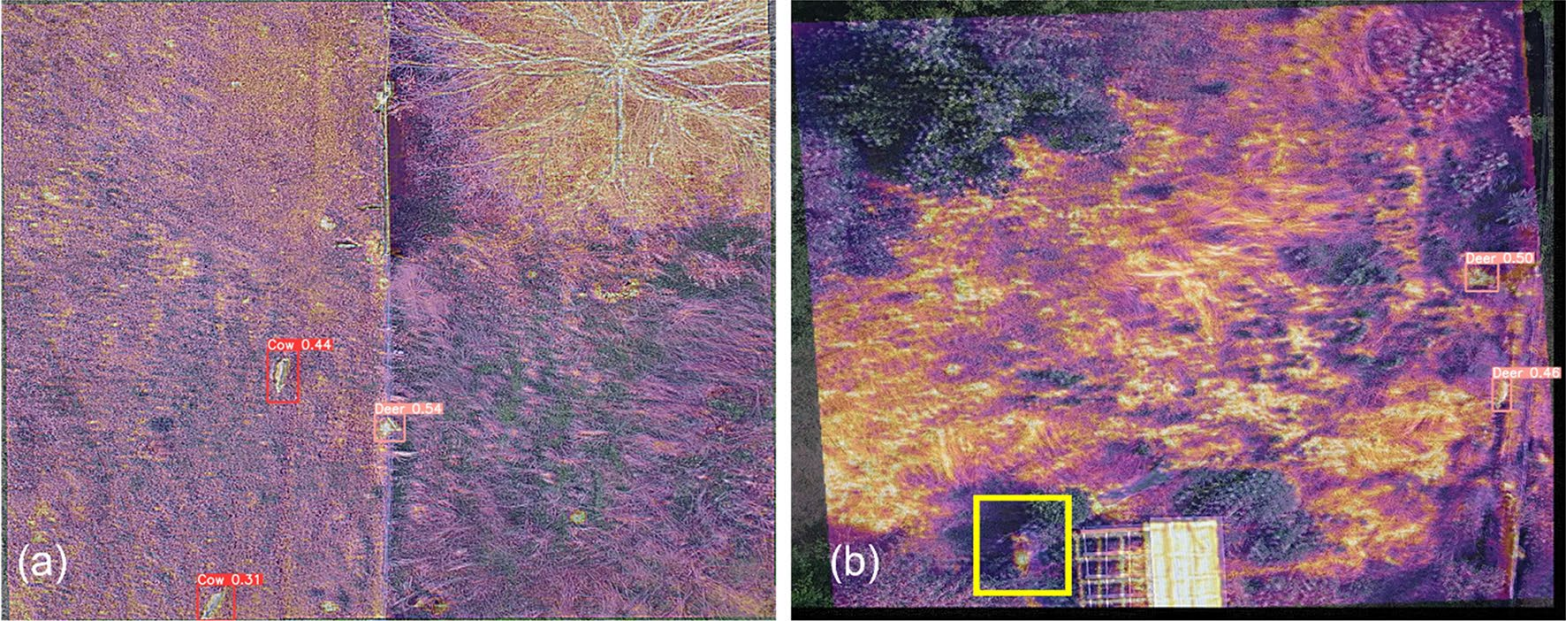
Examples of false positives regarding misclassification (**a,b**), and false negatives as non-detection or target animal (**b**). Two of three white-tailed deer (*Odocoileus* virginianus) were misclassified as cows (*Bos taurus*, **a**), and two hay bales were classified as cows (**b**, red boxes). The false negative occurred when the target animal (white-tailed deer, yellow box) was not detected (**b**).

Metrics of classification accuracy were computed based on comparing classification of animal objects in images without bounding boxes by architectures after training to original images containing bounding boxes that were manually drawn (ground truth) during image processing. We computed precision, recall, and mean average precision (mAP50; an additional measure of accuracy) as performance metrics of classification. Mean average precision (mAP) measured the correctness of animal detection (i.e., bounding box around animal object) and animal classification (i.e., species identification) for objects annotated by architectures in the test image dataset. Greater mAP values indicate greater model accuracy in animal detection and classification. For mAP50, a 50% threshold was considered for intersection over union (i.e., the overlap or intersection of predicted boundaries and actual animal boundaries; more information available in Supplementary Information Sect. 1.4). These metrics rely on three scenarios of correct or incorrect detection and classification to define^38^. Correct detections (draws a bounding box around the animal object) and classifications of target species (deer, cow, horse) by respective models (combinations of fusion methods and classification architectures) are defined as true positives. False positives occur when the respective model correctly detects (draws a bounding box around an object) but incorrectly classifies that object in an image as the target species, such as a different species (Fig. 3a) or inanimate object (Fig. 3b). False negatives occur when the respective model does not detect an individual of the target species when it occurs in an image (Fig. 3b). Accordingly, precision measures the proportion of true compared to false positives that the model correctly predicted, calculated as:

1 $$Precision=\frac{true \, positives}{true \, positives \, + \, false \, positives}$$

Recall measures the proportion of true positives compared to false negatives that the model correctly predicted, calculated as:

2 $$Recall=\frac{true \, positives}{true \, positives \, + \, false \, negatives}$$

Typically, overall accuracy considers false positives and negatives and is often defined as true positives divided by the sum of true positives, false positives, and false negatives (e.g.^14^). Similarly, we evaluated accuracy by comparing (1) human-drawn bounding boxes containing correctly classified animals (ground truth) to (2) objects in a bounding box automatically drawn and classified from respective model output (predicted). However, models did not necessarily draw the bounding box to entirely encompass the animal. Thus, models required a threshold of the number of overlapping pixels to evaluate if the bounding box adequately captured the animal object compared to the manually drawn, correct classifications (e.g., 60%, 80%, overlap with true positives; see Supplementary Information Sect. 1.4 online for additional information regarding intersection over union). Accordingly, we used mAP50 (mean average precision with a threshold overlap of 50%) as an alternative but accepted metric of accuracy, because it accounted for precision and recall while computing an average value for the overlap of predicted and ground truth bounding boxes for a range of values^38^. To further evaluate fusion methods and compare them to visible and thermal results alone, we ranked results for each fusion method with visible and thermal results using mAP50 for each animal species and classification architecture. We then summed the rank scores (1–10) among species and architectures, using the lowest score to determine the best performing fusion methods in context of visible and thermal results.

## Results

Between architectures, YOLOv5 (Table 2) outperformed YOLOv7 (Table 3) overall among metrics and animals in visible and thermal images, as well as among fusion methods. Although mAP50 for YOLOv7 was poor overall, we report results for both architectures to demonstrate that some fusion methods provided improvement for animal classification beyond visible and thermal results alone.

**Table 2:** Classification accuracy metrics of domestic cattle (*Bos taurus*), white-tailed deer (*Odocoileus virginianus*), and domestic horses (*Equus caballus*) for visible, thermal, and eight fusion methods for YOLOv5 learning module from images taken by a drone (unoccupied aircraft system or UAS).

Class	Classification accuracy										
	Metric	Visible	Thermal	GF	LP	SVD	Sparse	Gradient	TVM	WL + Swarm	WL + TVM
Cow	Precision	0.88	0.73	0.79	0.85	0.58	0.87	0.77	0.81	0.84	0.72
	Recall	0.75	0.75	0.75	0.80	0.14	0.80	0.66	0.85	0.84	0.65
	mAP50	0.89	0.77	0.83	0.88	0.16	0.93	0.69	0.84	0.86	0.74
Deer	Precision	0.72	0.81	1.00	0.74	1.00	0.72	0.77	0.61	0.73	0.52
	Recall	0.44	0.56	0.44	0.63	0.00	0.67	0.56	0.86	0.72	0.78
	mAP50	0.63	0.64	0.56	0.66	0.01	0.72	0.69	0.77	0.70	0.62
Horse	Precision	0.95	0.93	0.89	0.93	0.67	1.00	0.92	0.87	0.90	0.93
	Recall	0.93	0.92	1.00	0.93	0.87	0.93	0.77	0.87	0.87	0.88
	mAP50	0.99	0.97	1.00	0.95	0.89	0.99	0.95	0.95	0.95	0.96

*GF* guided filter method, *LP* Laplacian method.

**Table 3:** Classification accuracy metrics of domestic cattle (*Bos taurus*), white-tailed deer (*Odocoileus virginianus*), and domestic horses (*Equus caballus*) for visible, thermal, and eight fusion methods for YOLOv7 learning module from images taken by a drone (unoccupied aircraft system or UAS).

Class	Classification accuracy										
	Metric	Visible	Thermal	GF	LP	SVD	Sparse	Gradient	TVM	WL + Swarm	WL + TVM
Cow	Precision	0.26	0.35	0.53	0.38	0.16	0.54	0.41	0.00	0.32	0.37
	Recall	0.45	0.65	0.62	0.45	0.25	0.75	0.70	0.00	0.54	0.80
	mAP50	0.26	0.48	0.49	0.37	0.10	0.59	0.55	0.00	0.47	0.60
Deer	Precision	1.00	0.55	0.99	0.78	0.00	0.68	0.52	1.00	0.67	0.33
	Recall	0.00	0.55	0.44	0.77	0.00	0.33	0.67	0.00	0.57	0.50
	mAP50	0.00	0.51	0.50	0.85	0.00	0.37	0.62	0.00	0.44	0.46
Horse	Precision	0.26	1.00	0.46	0.50	0.00	0.41	0.54	0.80	1.00	0.70
	Recall	0.87	0.27	0.47	0.40	0.67	0.80	0.47	0.53	0.27	0.47
	mAP50	0.53	0.47	0.44	0.44	0.24	0.64	0.54	0.52	0.50	0.54

*GF* guided filter method, *LP* Laplacian method.

### Object quality

**Figure 4:**
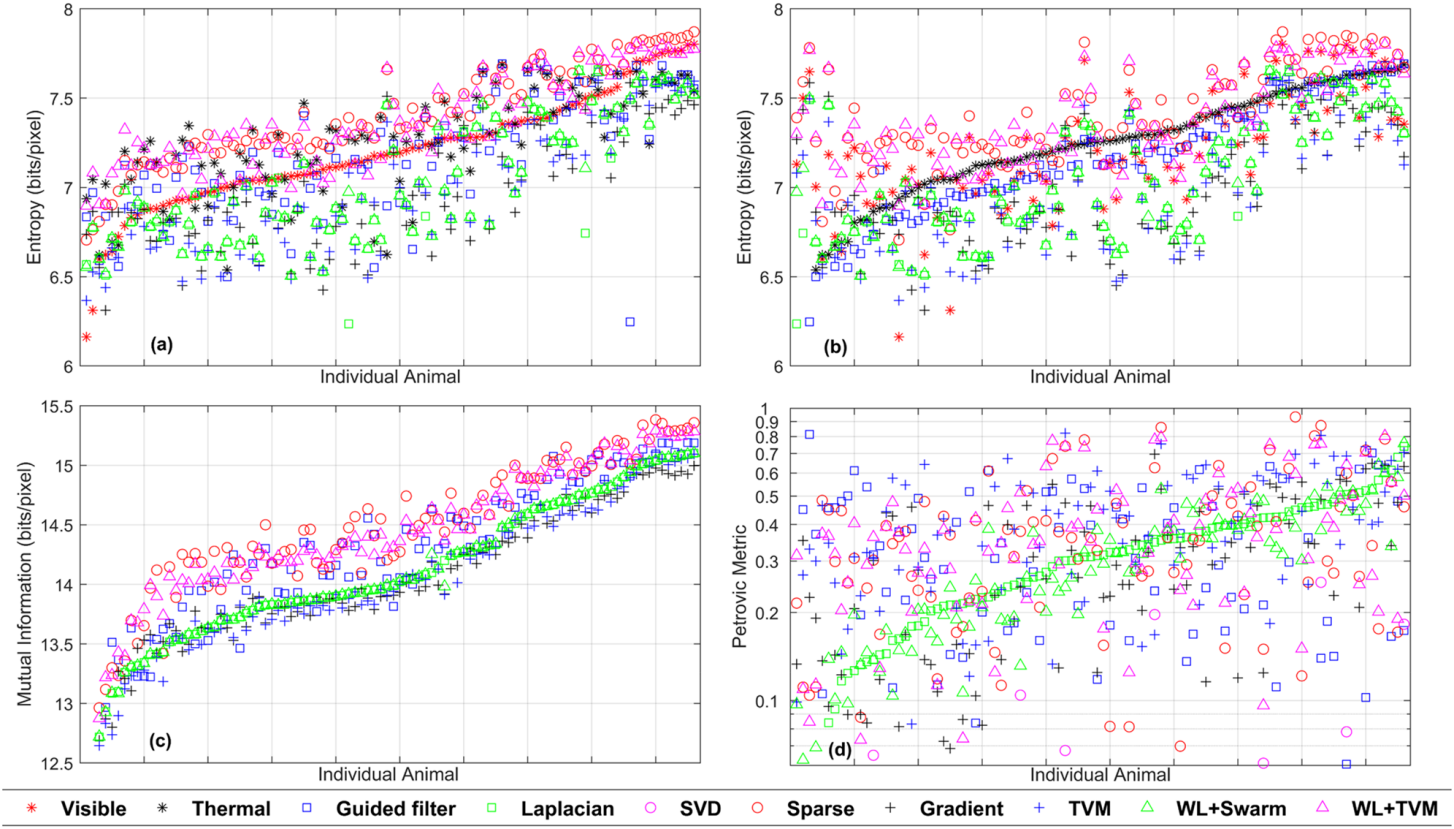
Plots of four metrics of image quality for 96 animal objects of domestic cattle (*Bos taurus*, cow), white-tailed deer (*Odocoileus virginianus*), and domestic horses (*Equus caballus*) in drone (unoccupied aircraft system or UAS) images automatically detected and classified using visible, thermal, and fused images produced by eight fusion methods. Animal objects on the x-axis do not correspond to the same individuals in each plot and are ordered in each plot to aid in interpreting the relative performance of fusion methods. Entropy values (bits/pixel), a measure of object information compared to the background, are ordered from descending to ascending values based on visible values (**a**) and thermal values (**b**). Values for mutual information (bits/pixel), a measure of the amount of information transferred from an individual image (visual or thermal) to the fused image (**c**), and the Petrovic metric (**d**), a measure of edge-preservation from 0 to 1 (unitless, shown on log scale), are ordered from descending to ascending values based on the values of a middle-ranked fusion method for this metric (Laplacian) and do not contain values for visible or thermal images.

Metrics of image quality for entropy indicated that Sparse and WL + TVM consistently provided more information than visual (Fig. 4a) and thermal (Fig. 4b) alone, indicating these fusion methods better characterized the animal object compared to the background than unfused images or other fusion methods. For cows and horses, Sparse had the highest entropy values in 54.5–75.0% of animal objects, respectively, compared to 18.8–22.7% of animal objects for WL + TVM. For deer, in contrast, WL + TVM had the highest entropy values (70.0% of deer objects) compared to Sparse (20.0%). Sparse, WL + TVM, and Guided filter had consistently high values for mutual information (Fig. 4c), indicating they transferred more information from visible and thermal images to fused images. Like entropy results, Sparse had the greatest values of mutual information for cows and horses (54.5–75.0% of animal objects, respectively) versus deer (20.0%) compared to WL + TVM (cows,18.8%; horses, 22.7%; deer, 80.0%). The Guided filter method performed best for mutual information in 6.8% of cow objects, 9.4% of horses, and no deer. Results for the Petrovic metric were approximately the same for all three animal species, and all but the SVD method performed similarly well (Fig. 4d), indicating consistent preservation of edge information of animal objects among fusion methods. The following four fusion methods had the greatest values for the Petrovic metric for all three animal species: TVM (34.4–40.0% of animal objects), Guided filter (25.0–27.3%), WL + TVM (15.0–15.9%) and Sparse (9.1–15.6%).

### Object detection and classification

Classification accuracy (mAP50) of cows for YOLOv5 was 16% higher in visible compared to thermal images (Table 2). Only Sparse improved overall accuracy beyond the performance for visible images alone (Table 2). In contrast, for YOLOv7, classification was about 85% better in thermal compared to visible images (Table 3). Classification improved over thermal images with three fusion methods (WL + TVM, 25%; Sparse, 23%; Gradient, 15%), and minimally with Guided filter (2%, Table 3).

For deer, classification accuracy was similar for visible and thermal images with YOLOv5 (i.e., < 2% increase from visible to thermal; Table 2). Four fusion methods provided considerable improvement (TVM, 21%; Sparse, 13%; WL + Swarm, 10%; Gradient, 9%), and one method (LP, 4%) provided minimal improvement compared to visible and thermal results (Table 2). For YOLOv7, visible was never accurate but increased fivefold for thermal (Table 3). Fusion improved classification of deer substantially over thermal results with the LP (67%) and Gradient (13%) methods (Table 3).

Classification accuracy of horses was near 100% for both visible and thermal images for YOLOv5 (Table 2). Among fusion methods, only Guided filter improved results beyond visible results (Table 2), although the gain was minimal (1% for visible, 3% for visible). For YOLOv7, visible improved accuracy 13% compared to thermal (Table 3). Among fusion methods, Sparse provided substantial gains in overall accuracy (21% improvement to visible), whereas Gradient and WL + TVM provided minimal (< 2%) gains compared to visible results (Table 3).

Rankings based on overall accuracy (mAP50) for animal classification among animals and architectures for fusion methods indicated that Sparse was the highest-ranking fusion method (Table 4), with the lowest rank score (15), followed by Gradient (25). The LP and WL + TVM methods ranked equally (28), followed by WL + Swarm (29), just above the rank score for visible and thermal results (30). Guided filter (31) and TVM (33) ranked below visible and thermal results (Table 4). SVD (51) was consistently poor and typically ranked last in most tests (Table 4).

**Table 4:** Ranking of animal classification results based on overall accuracy (mAP50) for domestic cattle (*Bos taurus*, cow), white-tailed deer (*Odocoileus virginianus*, deer), and domestic horses (*Equus caballus*, horses) for visible, thermal, and eight fusion methods for YOLOv5 and YOLOv7 learning modules from images taken by a drone (unoccupied aircraft system or UAS).

Rank	YOLOv5			YOLOv7		
	Cow	Deer	Horse	Cow	Deer	Horse
1	Sparse	TVM	Guided filter	WL + TVM	Laplacian	Sparse
2	Visible	Sparse	Visible (2)	Sparse	Gradient	Gradient (2)
3	Laplacian	WL + Swarm	Sparse (2)	Gradient	Thermal	WL + TVM (2)
4	WL + Swarm	Gradient	Thermal	Guided filter	Guided filter	Visible
5	TVM	Laplacian	WL + TVM	Thermal	WL + TVM	TVM
6	Guided filter	Thermal	Laplacian (5)	WL + Swarm	WL + Swarm	WL + Swarm
7	Thermal	Visible	Gradient (5)	Laplacian	Sparse	Thermal
8	WL + TVM	WL + TVM	TVM (5)	Visible	Visible (8)	Guided filter (7)
9	Gradient	Guided filter	WL + Swarm (5)	SVD	SVD (8)	Laplacian (7)
10	SVD	SVD	SVD	TVM	TVM (8)	SVD

Numbers in parentheses indicate ties in rank for corresponding numbers and methods.

## Discussion

Our results further promote fused thermal and visible imagery for improved detection and classification of animals in drone imagery as initially explored in two previous studies^17,28^. Broadening past approaches, we found that some fusion methods increased both image quality and classification metrics consistently over thermal and sometimes visual results alone, but these results differed by animal species. For deer, the most accurate fusion methods substantially increased classification accuracy over visible and thermal images alone. However, the most accurate fusion methods provided little improvement over classification of cows and horses from visible images alone. These differences are likely explained by the contrasting search images of the animals we surveyed, suggested in two previous studies^17,28^. Cows and horses were typically conspicuous in visible images compared to deer, which were more cryptic against their respective backgrounds and required additional thermal information for classification. Our results suggest that for cryptic species such as deer, the fusion of information in thermal and visible images improves classification over either image type alone. Understanding the tradeoffs in using fusion compared to visible images alone for automated animal classification can improve the results and efficiency of drone surveys among animal species that differ across a gradient of conspicuous to cryptic against their respective backgrounds.

Intrinsic (e.g., animal size, color, and shape) and extrinsic (e.g., image background and shadows) factors can influence accurate automated classification of animals^7,8,17,28,39^. If size, shape, color, or a combination of these or other features are distinctive, visible images often contain most of the identifying characteristics needed to accurately identify animal species, at least during diurnal periods with adequate lighting^8,17,28,39^. Cows and horses in our study represented large-bodied mammals with distinctive body shapes and colors against open pastures or contrasting color backgrounds. Larger animals in images comprise more pixels than smaller animals at the same ground sampling distance (GSD), providing more information and often better classification performance for automated approaches^28,39^. Similarly, distinct differences in body shape or appendages (e.g. beaks, hooves, antlers, etc.) can also provide information used to automatically classify animals to species^28,39^. Color contrast with background environments, like our black and brown cows and horses against a green pasture has also been shown to improve automatic detection and classification of animals^7,8,17,28,39^. A combination of the above factors likely explains our findings for cows and horses, where fusion methods provided minimal, if any, gains in classification performance compared to visible results alone.

In many natural situations encountered during surveys using visible imagery, animals have little to no contrast with surrounding environments , are partially obstructed, or occur in low light conditions^6,17,28^. In such cases, thermal images provide critically important complementary information needed for detection or classification of species, such as animal heat signatures against typically cooler ambient backgrounds^6,7,17,28^. None of the deer in our images were obstructed, but many (~ 60%) occupied shadowed areas. Thermal images alone provide little classification information for animals of similar sizes absent distinct shape features, such as large mammal species in our study. This lack of information is particularly evident in drone images recorded at higher flight altitudes because animals typically appear as indistinct color clusters against the ambient background and provide few distinguishing features^28^. Hence, our observed poor classification results for thermal images alone compared to visible results alone or fused results. Similarly, automated detection and classification of 5 Gy wolves (*Canis lupus*) and 6 fallow deer (*Dama dama*) in zoo enclosures was more difficult, due to their cryptic pelage against respective backgrounds and similar sizes, compared to 4 American bison (*Bison bison*) and 3 elk (*Cervus canadensis*), which were larger and more conspicuous^28^. Meanwhile, fusion of the two image types helped to improve classification for cryptic or shadowed species over visible or thermal alone in our study, resulting in an increase in performance for fused images from15–85% for deer, far exceeding fusion results for self-driving cars during daytime (5% better than visible alone and 29% better than thermal alone^26^).

Our study highlights some important methodological and computational constraints, strengths, and potential future improvements. Computing resources limited the maximum batch size for YOLOv7, which likely explained poorer results for this classification architecture compared to YOLOv5. Using larger batch sizes can increase performance for YOLOv7^40^ and is a likely next step for future research. Similarly, future studies could test fusion methods in other classification architectures such as CNNs^6,13–15^ and deep residual networks^13^.

Sparse and WL + TVM fusion methods performed consistently best for metrics of image quality, but these results translated to improved classification of animals only for Sparse, the top-ranking method by far. In contrast, WL + TVM ranked slightly better than either visible or thermal results for all three species. In our study, we trained our models to consider all three species simultaneously in non-annotated images to correctly detect target species, classify them, and exclude detecting or confusing them with other non-target objects. For studies of animals automatically classified from drone images, our fusion results represent an improvement compared to classifying a single species^6,7,14,15^, or multiple species limited to annotated boxes where the animal is already detected but not classified^13^. Our methodology and results also extend the utility of fusion approaches for drone imagery beyond detection of single species^17^ and classification among species with a few individuals present in the image^28^. Among fusion methods in our study, Sparse performed consistently well across two classification architectures, as well as three image quality and three classification metrics for three mammal species (one cryptic, two conspicuous). Future research could test Sparse performance with other image fusion methods^26^.

Our results demonstrate that image fusion is a viable option when images are limited (43–68 images of 77–265 animal objects in our study) for automated and accurate animal classification taken from visible and thermal drone sensors. Studies for other computer vision methods of animal classification from drone images used much larger numbers of images including > 900 images for koalas (*Phascolarctus cinereus*)^6^, and > 2000 tiles from image mosaics for caribou (*Rangifer tarandus*)^14^. However, increasing the number of training and testing images could also improve fusion results compared to the relatively few images collected for our study, as increasing the number of pre-classified images available to train models typically leads to better performance^38^. One solution is to use open-source repositories of pre-annotated objects, which provide large numbers of images and benchmark datasets for training and standardized comparisons across studies for other fields (e.g. ImageNet^18^). Such open-source, collaborative repositories for drone images of wild animals could advance automated classification for a variety of animal species; however, to our knowledge, only two such repositories are currently available (OUR^14^, AWIR—this study), Benchmark datasets for animals in drone images would benefit from high variation in image backgrounds, animal positions, group sizes, species, color, and other features, each of which typically improves performance of classification models, as demonstrated for camera trap studies^41,42^.

Our fusion results also are indicative of the benefits of employing drones capable of collecting visible and thermal images simultaneously when conducting animal surveys. Classification with fusion methods will yield the best results when the survey maximizes information provided by both visible and thermal sensors. Accordingly, characteristics of target animal species, environment, and time of day are critical considerations. Surveys that target animals that are distinct in size, shape, color, and background contrast relative to each other, will provide the most information for accurate detection and classification in visible images^13,28,39^. Endothermic compared to ectothermic animals will typically provide the most heat contrast of body compared to ambient temperatures in thermal images, unless ambient temperature is high^6,7,15,17^.

For visible images, conducting surveys at midday can minimized potential effects of shadows, which can hide or confuse detection in these images^43,44^. In contrast, in some instances shadows can enhance detection^10^. However, activity for endothermic animals is often greater in crepuscular periods, which could improve detection, but might also cause errors in double-counting animals^14,45^. For thermal surveys, early mornings provide the coolest temperatures compared to other times of day, even in warm environments where the image background approaches or exceeds the surface temperatures of endothermic animals^6,7,15,17^. Thus, conducting surveys in the morning in warm environments will likely maximize the benefit of heat contrast with target endothermic animals for thermal images and detection of these animals in visible images. If shadows do not enhance detection^10^, our results indicate that the fusion of both image types will offset potential drawbacks in decreased animal detection in shadows of visible images due to gains in information from fusing visible with thermal images leading to improved classification accuracy. Other image processing possibilities, particularly targeting ectotherms, include incorporation of algorithms utilizing color correlation measurements found in some camera trap systems (e.g.^46^). In other environments or seasons where the contrast between ambient temperatures and animal body temperatures are high, time of day may not be important for surveys or may be dictated by the constraints of animal behavior or other logistics. Additionally, using higher-resolution sensors or flying drones at lower altitudes can improve classification results or permit accurate classification of smaller animals^11,39^. Our results indicate that fusion methods are promising to advance automated detection and classification of animals from drone surveys, particularly for cryptic animals.

## Supplementary Information

Supplementary Information.

## Data Availability

Imagery collected and analyzed are available as unprocessed image pairs (with EXIF) in the Aerial Wildlife Image Repository (https://projectportal.gri.msstate.edu/awir/). Code developed to generate Fusion modules will be made available in Github. Classification architectures were cloned from https://github.com/ultralytics/yolov5/releases/tag/v6.1 for YOLOv5 and https://github.com/WongKinYiu/yolov7 for YOLOv7.
