# Supplementary material for: Fusion of visible and thermal images improves automated detection and classification of animals for drone surveys
Source: Sci Rep. 2023 Jun 27;13:10385. doi: 10.1038/s41598-023-37295-7 (PMC10300091; doi:10.1038/s41598-023-37295-7)
Supplement: Supplementary file 1 — Supplementary Information. [file 41598_2023_37295_MOESM1_ESM.docx]

**Supplemental Information**


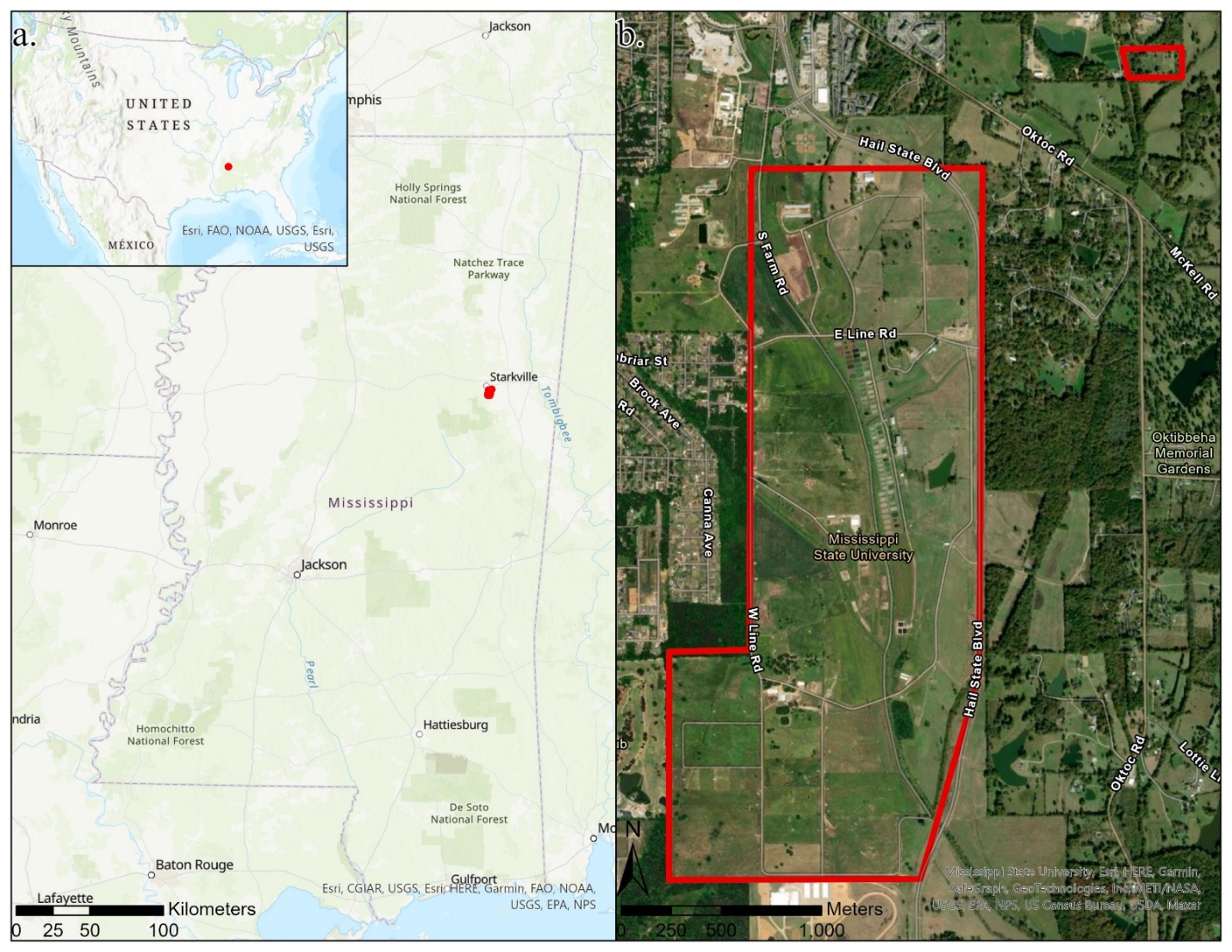


**Figure S1.** Mississippi Agriculture and Forestry Experiment Station H. H. Leveck Animal Research Center study site in Mississippi, USA (a), where images were collected from a drone (unoccupied aircraft system) at the South Farm of Mississippi State University (b) over areas outlined in red, including pastures (middle block) for domestic cows (*Bos taurus*) and horses (*Equus caballus*) and captive pens (northeast block) for white-tailed deer, *Odocoileus virginianus*).

**Supplementary methods for image registration, fusion, classification, and evaluation procedures**

**1.1** **Image Registration**

Sharma and Thé 2013^1^ detailed the procedure for automatically registering images with MATLAB^®^, based on several studies (e.g.^2–4^). The DJI XT2 sensor captured visible images of 4000×3000 pixels and thermal images of 640×512 pixels. Using Exiftool^5^, we obtained the time, GPS, exposure, aperture, shutter speed and other details of captured images, in addition to altitude, yaw, pitch, and roll of the drone platform. Because the scaling, translation, and rotation options available on standard Exiftool packages did not directly help in registering the thermal image to the corresponding visible image, we used the gimbal yaw parameter to first rotate both images. Then, we applied an interpolation using Lanczos kernel-based image scaling^6^ to obtain a scaled image. Last, we translated the scaled thermal image onto the visible image canvas to obtain the overlaid images (i.e., fused image). To obtain the precise translation and scaling factor, we aligned a set of prominent edges in the images^4,7^. We chose the most distinguishable straight-crossing edges (e.g., distinct barricade walls in the deer enclosure images, trail paths in cow images) and long edges with curves spanning >80% of length (e.g., entire border of a pond) of a set of six image pairs (thermal and visible) to match images with the minimum amount of error. Six images were an arbitrary representative set of images. We then manually checked translation vectors for each pair of images to verify image registration. The best choice of scaling factor was 2.8 (to the thermal image) after the centers of the two images were aligned, but because of changes in the yaw between image pairs and drone movement, a correction detail to each image pair's translation (< 20 pixels) was manually evaluated for each image pair. After scaling the thermal image and translating the thermal image onto the visible image, the resulting intersection canvas size was 1792 × 1434 pixels in the 0° yaw case. Images converted to other sizes, either because of rotation effects or different original canvas size, were converted to this size as a standard for ease of processing. We observed later that a re-run of registration algorithms on a slightly larger image than the intersection region can substitute our manual process. In summary, we injected information from the resampled smooth thermal bands into the sharper components from the visible image after suitable processing (i.e., registration, scaling, transformation, etc.). Fig. S2 provides an example case of the fused image after a failure in registration. Copies of fence walls were generated after fusing and neither of the two object copies were better than the individual images because of the image combining using irrelevant data.

**
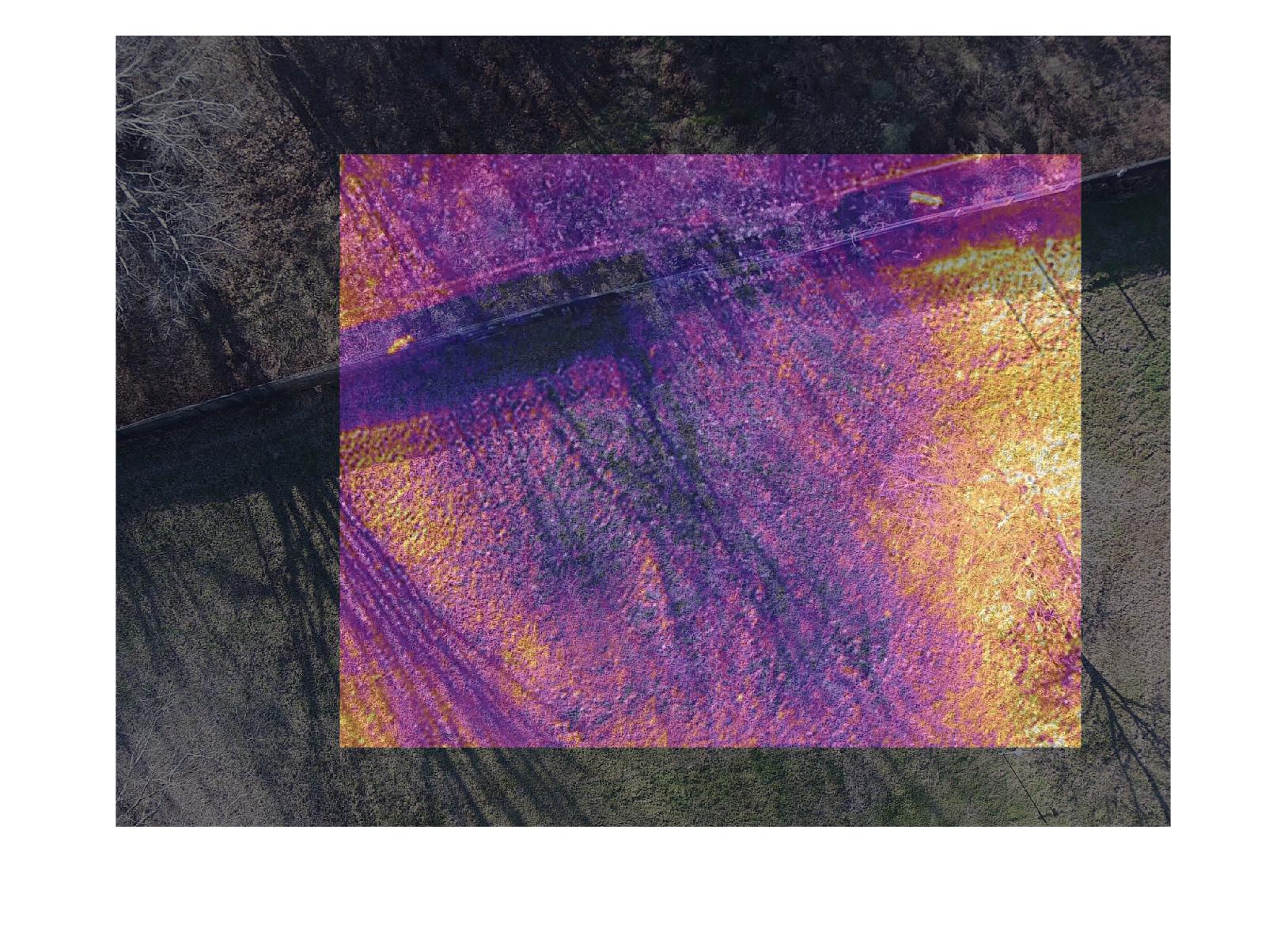
**

**Figure S2.** Error in registration of a fused image after improper registration, showing repeated objects (e.g., fence line).

- 1. **Image Fusion methods**

We compared eight fusion methods. We used three multiresolution-based methods. In the Guided filter method, a block in the fused image was obtained as the sum of an affine transformation of the visible image where the coefficients were a function of the corresponding block’s pixels from the thermal image, chosen to satisfy a local mean square linear regression problem^8^. The Laplacian/Gaussian pyramid (Laplacian) method generated three levels of the image’s Laplacian pyramid^9^. Then, we represented each image block using a dictionary based on matching pursuits algorithm. Finally, we considered local variability of the coefficients in each block to determine the coefficients for the convex sum of the visible and thermal image blocks that created the fused image. The procedures based on Laplacian pyramids or guided filters operate on preserving or improving the information content in the high pass/edge region. The singular value decomposition (SVD) method generated an image representation based on the SVD of the image pixels. Following Naidu 2011^10^, we divided the input image into k×k (k = 4) non-overlapping blocks of pixels. Each block matrix was vectorized and concatenated to form a fat matrix (denoted as X) of k rows. The eigen vector matrix of XX`, denoted as U, was then used to obtain the representation U`X, which had its dominant eigen value row as approximations (low-pass terms) while the other k-1 (corresponding to the lower eigenvalues) were detail coefficients. This was not very helpful because this method caused a lot of edge effects.

The sparsity-based fusion method (Sparse) represented the low-pass (approximation) coefficients using a sparse vector and used different combining rules for the high pass and low pass coefficients^11^. The reconstruction was standard. First, each block of each image obtained the sparse representation using an L_1_ minimization and choosing the best sparse representation within 100 iterations using the orthogonal matching pursuit algorithm as in Wang et al. 2020^9^ and Naidu 2011^10^. From the visible and thermal representations, we chose the one with the largest L_1_ norm as the ‘fused’ sparse domain representation for the low-pass coefficients, and max-absolute values for high-pass coefficients. Finally, we reconstructed the block using the dictionary (DCT, DWT).

We used two optimization-based methods that differed regarding their objective functions. For the gradient method (Gradient), we used a simple one-pixel difference map (gradient map), owing to its better spatial localization than a traditional derivative operator, which also allowed us to retrieve the original image when down-sampling was not used^12^. The total variation distance (TVM) method was another local optimization approach that solved a block-wise optimization problem to find the best pixel intensities that reduced a weighted distance/Lagrangian between images, thus preserving edges^13,14^.

The first of two hybrid approaches, wavelet & TVM (WL+TVM), generated one low pass and two high pass outputs using a double density wavelet transformation (i.e., equivalently a three-channel perfect reconstruction filter bank). From the three outputs of the thermal and visible images, the algorithm calculated: 1) weights for linear combinations of the coefficients, 2) a threshold to cap the detail present in one of the two images, and 3) the depth of transformation. The objective function used was a linear function of the structural similarity metric between fused and unfused images (e.g., fused vs. visible and fused vs. thermal^15^). The wavelet & Swarm (WL+Swarm) method performed a dual-tree discrete wavelet transform on the visible and thermal images, and the optimization problem chose the linear combining weights that ensured highest entropy of the block^16^.

**1.3 Object Detection and Classification Framework**

YOLO (acronym for “You Only Look Once”) is a popular network structure for real-time object detection tasks^17^. We used YOLOv5 and YOLOv7 in our experiments. For implementation, we cloned YOLOv5 from <https://github.com/ultralytics/yolov5/releases/tag/v6.1> and YOLOv7 from <https://github.com/WongKinYiu/yolov7>. Details of network architectures and block diagram representations can be obtained from Xu et al. 2021^18^ for the YOLOv5 network, and Ren et al. 2021^8^ for the YOLOv7 network. A stride size of 32 pixels was configured (i.e., ratio the algorithm uses to down-sample the input), and both learning engines padded the images to 1792 × 1440 for processing. The 6-pixel column addition was part of the networks’ pre-processing and only affected a few of the corner objects, if ever. Tuning the hyperparameters and adjusting the model architecture, users can achieve a trade-off between detection accuracy and computational efficiency based on their specific use cases. The hyperparameters we used were a learning rate of 0.01 kept constant at the start and end, a momentum parameter for ADAM optimizer of 0.937, image augmentation degrees of 0 to avoid image rotation with the setting for other image pre-processing options (e.g., scaling, shear, flip, translation) and all HSV augmentation fractions kept unchanged from default settings (hue = 0.015, saturation = 0.7 and value = 0.4) Between YOLOv5 and YOLOv7, we used 100 and 135 epochs and 16 and 12 batch sizes, respectively. For both algorithms we used a pixel stride of 32 pixels and an IOU threshold of 0.2.

**1.4 Evaluation Criteria and Metrics**

To evaluate the performance of fusion methods for improving animal object detection and classification, we considered the following objective image block-based indices to assess animal object quality: entropy, mutual information, and the Petrovic metric. We also considered the following classification accuracy metrics: precision, recall and mean average precision.

Entropy reflects the actual information in the individual image^19^. Given a block of pixels, the entropy of the block was calculated using the normalized histogram of the pixel intensities in each of the three color dimensions as the (empirical) probability mass function in the standard formula. If *p(i)* denotes the discrete density of intensity *i*, then entropy is represented as

$$\sum p\left( i \right)log_{2}\left( p\left( i \right) \right)$$

(S1)

For a color image, as an approximation, we used the sum of the local entropy from each channel because the joint entropy of the three channels would involve computation of cross entropies across pairs and the joint information of the color-image entropy.

Mutual information (MI) represented the amount of information transferred from the individual image to the fused image. For images A and B, MI was calculated as

$$\sum_{i} \sum_{j} p_{\left\{ A, B \right\}} \left( i, j \right)\log\frac{p_{\left\{ A, B \right\}} \left( i, j \right)}{p_{\left\{ A \right\}} \left( i \right)p_{\left\{ B \right\}} \left( j \right)}$$

(S2)

The gradient based Petrovic measure represented the quality of edge preservation^12^. It was calculated as the perceptual loss of information in the fused image (F) from the individual source image (A), denoted as Q_A,F_. We calculated Q_A,F_ in two steps. First, we calculated edge strength and orientation using a Sobel operator. Second, let $g_{A}\left( x,y \right)$ and $d_{A}\left( x,y \right)$ respectively denote the resultant gradient amplitude and orientation at pixel location (x, y) of image A. Then we calculated the ratio (smaller / greater) of the gradients $g_{A},g_{F}$ and the absolute differences in the angles $d_{A},d_{F}.$ These two difference terms were supplied as parameters to a standard sigmoid, and the outputs were multiplied to get Q_F,A_(x, y). We used Q_F,A_(x, y) as a measure of edge preserving quality of the image after processing. In our case, we calculate Q_F,T_, Q_F,RGB_ and a weighted average of them to get the Petrovic measure Q_F,T,RGB_.

When using classification accuracy as a metric, the comparison is based on both the labels and bounding boxes of the animal objects obtained from the algorithm (predicted) and from manual labelling (ground truth). For the multi-class case, the definitions of true positive (TP), true negative (TN), false positive (FP) and false negative (FN) were defined for each class by grouping all other class results into one negative group and using the same formulae as in the binary case. For calculating precision, recall, and mean average precision (mAP50), we began by determining these metrics. For example, consider there were originally 7, 8, and 10 cow, deer and horses, respectively (Table S1). Animals that were not detected were grouped in a class called “Missed” for mimicking the true scenario such as animals in shadows (Fig. S3). Here the number of FP cow, deer and horses were 3, 1, and 1, respectively. The number of FN cow, deer and horses were 2, 3, and 3, respectively. Precision (or positive predicted value) was defined as TP / (TP + FP) and represented the proportion of predicted positives that was truly positive. Recall (or true positive rate or sensitivity) defined as TP / (TP+FN), answers which proportion of actual positives were correctly classified. For the example, the precision and recall values would be cow (5/8, 5/7), deer (4/5, 4/8), and horse (7/8, 7/10). There is a trade-off between recall and precision, and both values cannot be high for a given evaluation in real scenarios with FP and FNs. This balance depends on the value of the classifier threshold, which is used to predict a label to the detected object.


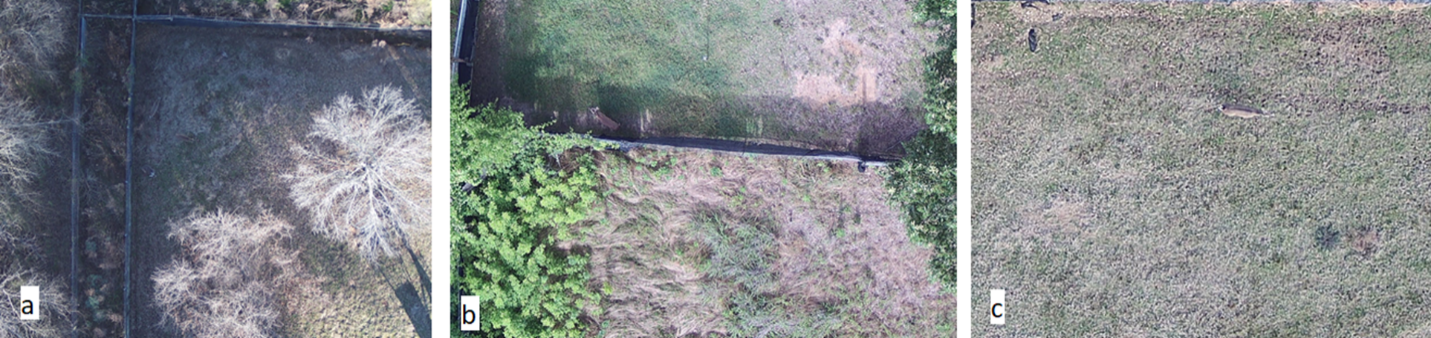


**Figure S3**. Aerial mages of white-tailed deer (*Odocoileus virginianus*) among three shadow situations: deer (*n* = 4) in shadows and not easily distinguishable (a), a deer in shadows but distinguishable (b) and a deer in the open (c).

Mean average precision (mAP) is a measure of both the correctness of the classification label and the correctness of the region detected as the object. It thus compares the ground-truth bounding box to the detected box. The higher the mAP, the more accurate the model is in its detections. Intersection over union (IoU) is the ratio of the number of intersecting pixels to the number of union pixels between the two (ground-truth and predicted) bounding boxes. IoU measures how much a predicted boundary overlaps with the ground truth. We considered an IoU threshold of 0.5. Therefore, we considered a prediction TP only if IoU > 0.5, a FP when IoU < 0.5, and a FN when a wrong classification occurred. As shown in the output images (Fig 3), each predicted bounding box was accompanied by a confidence value ($\in[0, 1])$, which was used to grade/rank this prediction. The average precision (AP50) for each class was calculated as the area under the precision-recall curve with a 50% (0.5) threshold for IoU. The mAP50 for object detection was the average of the AP calculated for all the classes. A high mAP50 indicated that the model was consistent among different confidence thresholds (i.e., it had good recall at higher confidence thresholds and good precision at lower confidence thresholds).

**Table S1.** Confusion matrix of example data representing drone (unoccupied aircraft system) image evaluations for cow, deer, and horses regarding ground truth animal numbers (Actual) and predicted numbers based on fusion method performance.

|  |  | Actual | | |
| --- | --- | --- | --- | --- |
|  |  | Cow | Deer | Horse |
| Predicted | Cow | 5 | 1 | 2 |
|  | Deer | 0 | 4 | 1 |
|  | Horse | 1 | 0 | 7 |
|  | Missed | 1 | 3 | 0 |

**References**

1. Sharma, G. & Thé, A. Automating Image Registration with MATLAB. *https://www.mathworks.com/company/newsletters/articles/automating-image-registration-with-matlab.html* (2013).

2. Liu, F. & Seipel, S. Infrared-visible image registration for augmented reality-based thermographic building diagnostics. *Vis. Eng.* **3**, 1–15 (2015).

3. Bulanon, D. M., Burks, T. F. & Alchanatis, V. Image fusion of visible and thermal images for fruit detection. *Biosyst. Eng.* **103**, 12–22 (2009).

4. Maurya, L., Mahapatra, P. & Chawla, D. A registration strategy from scale adjustment to fine for thermal-visible face images. *Infrared Phys. Technol.* **120**, 104001 (2022).

5. Exiftool. ExifTool. *https://exiftool.org/* (2022).

6. Aguilera, C. A. V. LanczosFilter. *https://www.mathworks.com/matlabcentral/fileexchange/14041-lanczosfilter-m* (2016).

7. Bilodeau, G. A., St-Onge, P. L. & Garnier, R. Silhouette-based features for visible-infrared registration. in *IEEE Computer Society Conference on Computer Vision and Pattern Recognition Workshops* (2011). doi:10.1109/CVPRW.2011.5981676.

8. Ren, L., Pan, Z., Cao, J., Liao, J. & Wang, Y. Infrared and visible image fusion based on weighted variance guided filter and image contrast enhancement. *Infrared Phys. Technol.* **114**, (2021).

9. Wang, Z., Cui, Z. & Zhu, Y. Multi-modal medical image fusion by Laplacian pyramid and adaptive sparse representation. *Comput. Biol. Med.* **123**, 103823 (2020).

10. Naidu, V. P. S. Image fusion technique using multi-resolution singular value decomposition. *Def. Sci. J.* **61**, (2011).

11. Liu, Y., Liu, S. & Wang, Z. A general framework for image fusion based on multi-scale transform and sparse representation. *Inf. Fusion* **24**, 147–164 (2015).

12. Petrovic, V. S. & Xydeas, C. S. Gradient-based multiresolution image fusion. *IEEE Trans. Image Process.* **13**, 228–237 (2004).

13. Du, Q., Xu, H., Ma, Y., Huang, J. & Fan, F. Fusing infrared and visible images of different resolutions via total variation model. *Sensors (Switzerland)* **18**, (2018).

14. Ma, J., Chen, C., Li, C. & Huang, J. Infrared and visible image fusion via gradient transfer and total variation minimization. *Inf. Fusion* **31**, (2016).

15. Guo, H., Chen, J., Yang, X., Jiao, Q. & Liu, M. Visible-Infrared Image Fusion Based on Double- Density Wavelet and Thermal Exchange Optimization. in *IEEE Advanced Information Technology, Electronic and Automation Control Conference (IAEAC)* (2021). doi:10.1109/IAEAC50856.2021.9390665.

16. Madheswari, K. & Venkateswaran, N. Swarm intelligence based optimisation in thermal image fusion using dual tree discrete wavelet transform. *Quant. Infrared Thermogr. J.* **14**, (2017).

17. Redmon, J., Divvala, S., Girshick, R. & Farhadi, A. You only look once: Unified, real-time object detection. in *Proceedings of the IEEE Computer Society Conference on Computer Vision and Pattern Recognition* vols 2016-Decem (2016).

18. Xu, R., Lin, H., Lu, K., Cao, L. & Liu, Y. A forest fire detection system based on ensemble learning. *Forests* **12**, 217 (2021).

19. Szeliski, R. Computer Vision : Algorithms and Applications 2nd Edition. *Springer* (2021).
